# Nomogram for predicting post-progression-free survival in patients with recurrent pancreatic ductal adenocarcinoma after radical surgery: a retrospective analysis

**DOI:** 10.3389/fmed.2024.1486750

**Published:** 2024-12-06

**Authors:** Dailei Qin, Pu Xi, Kewei Huang, Lingmin Jiang, Zehui Yao, Ran Wei, Shengping Li

**Affiliations:** State Key Laboratory of Oncology in South China, Guangdong Provincial Clinical Research Center for Cancer, Sun Yat-sen University Cancer Center, Guangzhou, China

**Keywords:** nomogram, recurrence, chemotherapy, pancreatic adenocarcinoma, post-progression-free survival

## Abstract

**Background:**

Radical resection is the only curative method for patients with pancreatic adenocarcinoma (PDAC). However, nearly 85% of PDAC patients suffer from local or distant recurrence within 5 years after curative resection. The progression of recurrent lesions accelerates the mortality rate in PDAC patients. However, the influence of clinicopathological factors on post-progression-free survival (PPFS), defined as the period from tumor recurrence to the timing of the progression of recurrent lesions, has rarely been discussed. The present study aimed to explore the independent prognostic factors for PPFS and construct a nomogram for PPFS prediction.

**Materials and methods:**

The 200 recurrent PDAC patients were divided into training and validation groups by leave-one-out cross-validation. The patients’ clinicopathological characteristics were compared through a chi-square test. Meanwhile, these factors were enrolled in the univariate and multivariate COX regression to find the independent prognostic factors of PPFS. Moreover, the Kaplan–Meier survival analysis based on the independent prognostic factors was performed. Finally, we constructed a nomogram model for PPFS prediction, followed by an effectiveness examination.

**Results:**

PDAC patients who received multi-agent chemotherapy after surgery showed a longer PPFS than the single-agent chemotherapy group. PDAC patients who received multi-agent chemotherapy after recurrence showed a similar PPFS compared to the single-agent chemotherapy group. Local recurrence with distant metastases, early recurrence, lympho-vascular invasion, higher T stage, and higher N stage predicted shorter PPFS in recurrent PDAC patients. Finally, a nomogram to indicate the progression of recurrent lesions was constructed.

**Conclusion:**

Multi-agent chemotherapy is recommended for PDAC patients after surgery. Meanwhile, single-agent chemotherapy also deserves consideration after tumor recurrence. Moreover, the nomogram could be used in PPFS prediction.

## Introduction

PDAC is the major component of pancreatic cancer (1, 2). As reported in previous research, the 5-year overall survival rate of PDAC is less than 10% (3). Moreover, PDAC was expected to become the second leading cause of cancer-related death by 2030 (4). So far, radical resection has been the only curative method for PDAC (5). Unfortunately, even though the adjuvant chemotherapy has been performed, nearly 85% of resected cases eventually experience tumor recurrence (6, 7). The relationship between the clinicopathological factors and tumor recurrence in PDAC has been explored previously. In detail, PDAC patients who received adjuvant chemotherapy achieved longer progression-free survival and overall survival compared to untreated patients (8, 9). However, the restriction effect of chemotherapy on recurrent PDAC has been rarely discussed.

The advancement of recurrent lesions was defined as follows: at least 20% increase in maximum diameter of the primary recurrent lesions, or detection of any new recurrent lesions in the distant tissue. Meanwhile, the period from tumor recurrence to the timing of the progression of recurrent lesions was defined as PPFS. The progression of relapse lesions reflects the weakness of the anti-tumor immune system and the production of circulating tumor cells, predicting poor prognosis (10–12). According to the NCCN guidelines, a change to an alternate chemotherapy regimen is usually recommended for recurrent PDAC patients. However, there lacked of a definite chemotherapeutic regimens for recurrent PDAC patients in the NCCN guidelines (13). Therefore, exploring independent prognostic factors of PPFS and further creating an analysis tool to predict the risk of PPFS is crucial for developing suitable adjuvant chemotherapy regimens for recurrent PDAC patients.

Several prediction models have been used to estimate the overall survival or progression-free survival of PDAC patients (14–16). Some independent factors in these models such as tumor markers or chemotherapy regimens were only collected before or soon after surgeries. The updated data after tumor recurrence were scarcely mentioned. Moreover, previous partial research only recorded whether PDAC patients received chemotherapy or not, the detailed classification of chemotherapy regimens has rarely been established. However, the PPFS of recurrent PDAC patients is greatly impacted by characteristics of relapse lesions rather than primary tumor features. Therefore, prior nomogram models may be less effective for PPFS prediction. Considering the lack of a specific predictive model for PPFS estimation, it was necessary to build a novel nomogram model for recurrent PDAC patients.

**Figure 1:**
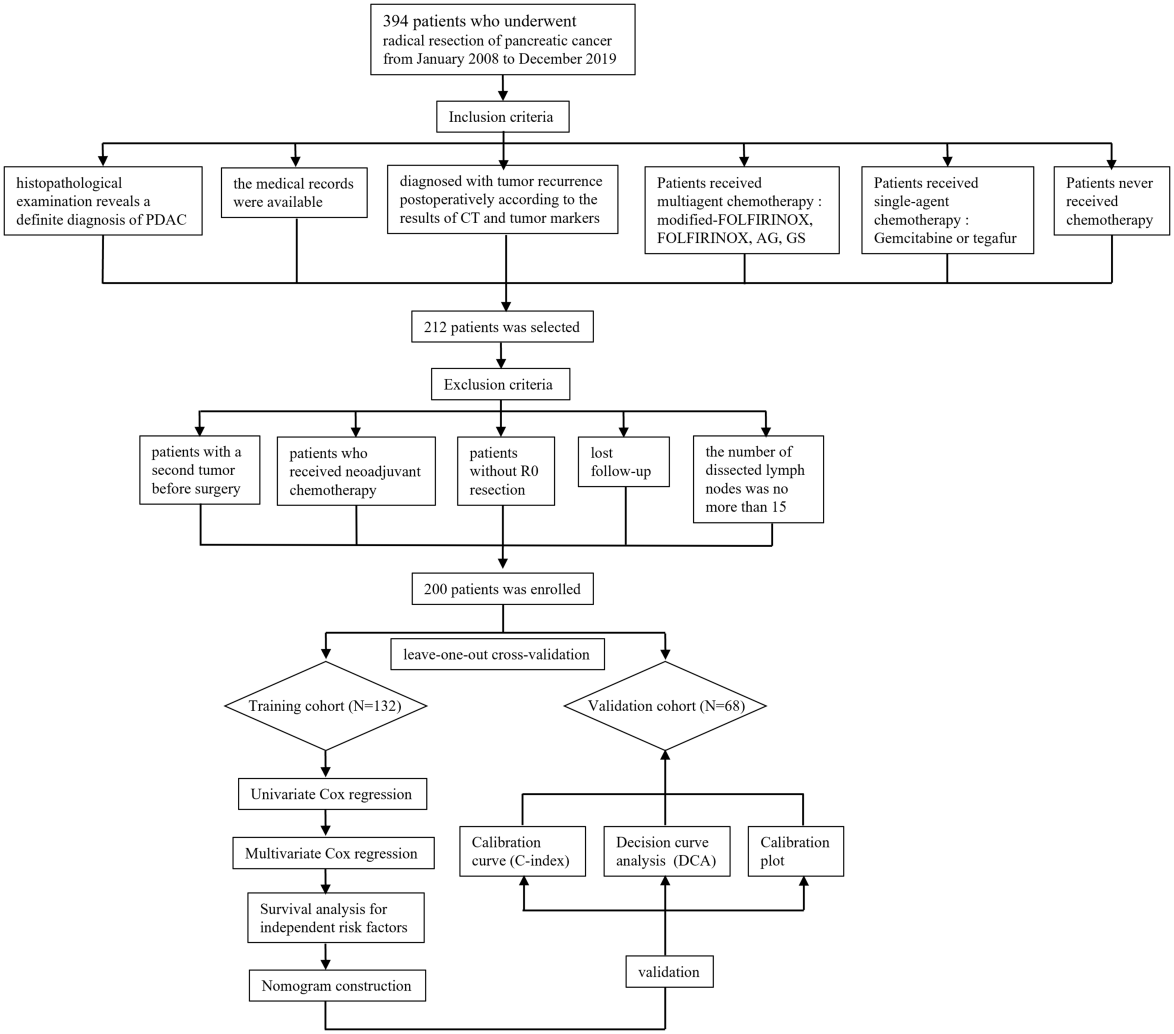
Flow chat of patients’ enrollment and study design.

In order to ensure the quality and reliability of data analysis, all participants were screened by their clinical and pathologic features. The flow chart has been established in Figure 1. Finally, a total of 200 recurrent PDAC patients were collected from 394 surgically resected cases with pancreatic cancer.

## Materials and methods

### Patient’s enrollment and grouping

A total of 394 patients who underwent radical resection for PDAC from January 2008 to December 2019 were collected from medical records (Figure 1). Pancreaticoduodenectomy and distal pancreatectomy are the main type of radical resection (17, 18). Tumoral resectability was investigated by a professional multidisciplinary team for PDAC based on imaging findings from computed tomography (CT), magnetic resonance imaging (MRI), and positron emission tomography-CT (PET-CT). Three chief physicians skilled in pancreaticoduodenectomy and distal pancreatectomy performed all of the surgeries included in this study. The attending clinician and resident physician were also required to participate in the surgical procedure. Furthermore, open, laparoscopic, and robotic surgery was chosen according to the clinical tumor state with consent obtained from patients.

The inclusion criteria concluded as follows: (1) histopathological examination reveals a definite diagnosis of PDAC, (2) diagnosed with tumor recurrence postoperatively according to the results of CT, tumor markers, and biopsy pathology, (3) the medical records were available, (4) Patients received multiagent chemotherapy: modified-FOLFIRINOX, FOLFIRINOX (oxaliplatin, Irinotecan, calcium folinate, fluorouracil), AG (nab-paclitaxel, gemcitabine), GS (gemcitabine, tegafur), (5) Patients received single-agent chemotherapy: Gemcitabine or tegafur, (6) Patients never received chemotherapy. On the contrary, the exclusion criteria were represented as follows: (1) patients with a second tumor before surgery, (2) patients who received neoadjuvant chemotherapy, (3) patients without R0 resection (the margin for R0 resection was described as 1.5–2 mm in the previous study) (19), (4) lost follow-up, (5) the number of dissected lymph nodes was no more than 15.

The patients enrolled in the present study were subsequently divided into training and validation group by leave-one-out cross-validation (LOOCV) method. In detail, one patient was enrolled into validation group each time, while the rest of patients served as a training group. Then, the accuracy of this model was recorded from analysis tool. The above procedure was repeated until all of the patients were tested as a validation sample. The final grouping schemes was determined by the average accuracy.

The consent to use the medical records has been obtained from all of patients enrolled in the present study. The Ethics Committee approved the retrospective study. This study was registered with www.researchregistry.com. This work has been reported according to the STROCSS (Strengthening the Reporting of Cohort Studies in Surgery) criteria (20).

### Collection of clinicopathological characteristics

The clinicopathological factors included in this research were chosen based on the results of previous prognostic analyses (9, 14). It is worth mentioning that some of the factors were acquired at the time points after tumor recurrence because the present study focused on the progression of relapse lesion in PDAC patients. In detail, the pathological diagnosis was acquired from experienced pathologists, including the tumor size, tumor differentiation, lymph node metastasis, microvascular invasion, lymph vascular invasion, and adjacent organ invasion. Moreover, several inflammation indices, such as the neutrophil-to-lymphocyte ratio (NLR) and platelet-to-lymphocyte ratio (PLR), were involved in this study. Besides, this study also registered clinical factors such as age, gender, serum levels of carbohydrate antigen 19-9 (CA19-9), and carcinoembryonic antigen (CEA) after confirmation of tumor recurrence. Moreover, the key features of tumor recurrence were also registered in this research, including time to recurrence (the cut-off value to define early and late recurrence was one year after surgery) and recurrence patterns (the definition of different relapse patterns referred from the research by Groot) (7, 21).

### Development and classification criteria of chemotherapy regimens

The chemotherapy regimens mentioned in the present study were applied after radical surgery or tumor recurrence according to the recommendation from NCCN (2021 Ver2.0) guidelines for PDAC respectively (13). Meanwhile, the patients’ will and Eastern Cooperative Oncology Group Performance Status (ECGO PS) were considered before developing the chemotherapy schema. The modified-FOLFIRINOX or FOLFIRINOX were the preferred option for PDAC patients in better physical condition or ECGO PS range from 0 to 1 point. If patients had a poor overall physical condition (ECGO PS range from 2 to 5 point), gemcitabine or tegafur will be recommended. AG or GS chemotherapy schema were usually applied in recurrent PDAC patients who were not sensitive to FOLFIRINOX chemotherapy schema. Furthermore, the chemotherapy regimens were divided into three levels through the variety of drug utilization. Firstly, the patients who never received chemotherapy after surgery or tumor recurrence were classified as “untreated.” Then, the patients who got only gemcitabine or tegafur therapies were defined as “single-agent chemotherapy.” Finally, the patients who underwent modified-FOLFIRINOX or FOLFIRINOX, AG, GS chemotherapy schema were categorized as “multi-agent chemotherapy.”

### Follow-up and outcome adjudication

The follow-up began at the time of tumor recurrence after radical surgery. The patients were recommended to undergo outpatient review every 3 months. Meanwhile, abdominal and chest CT, CA19-9, and CEA were performed regularly after surgery. If the outpatient review were unavailable for some patients, telephone contact would be the alternative method. The endpoint of the present study was progression in recurrent lesions, which is defined as follows: (A) ≥20% increase in maximum diameter of the primary recurrent lesions, (B) or detection of any new recurrent lesions in the distant tissue. The outcome adjudication was made after the imaging examination or pathology diagnosis during follow-up.

### Statistical analysis

The comparison of clinicopathological characteristics between the early and late recurrent groups was conducted using the chi-square test. The relationship between clinicopathological factors and PPFS was investigated using Kaplan–Meier methods. In detail, the log-rank test was utilized when the survival curve was not crossed, while the landmark analysis was applied when the survival curve was crossed. Multivariate Cox regression analysis was used to detect the independent prognostic factors for PPFS after completing the study of univariate Cox regression. The concordance indexes (C-indexes), calibration plots, and decision curve analyses (DCA) were utilized to compare the predictive ability between the nomogram and TNM-stage prediction models. A two-tailed *p* < 0.05 was considered statistically significant in the present study. All statistical analyses were conducted using SPSS software version 22 and R software version 4.2.2 (R Development Core Team; http://www.r-project.org). Moreover, the R packages “getsummary, tidyverse, survival, plyr, broom, forestmodel, ggplot2, rms, survminer, and ggDCA” were used in this research.

## Results

### Patient’s clinicopathological characteristics

A total of 394 PDAC patients received radical surgery between January 2008 and December 2019, while 212 cases of them were eventually diagnosed with tumor recurrence. Meanwhile, 12 recurrent PDAC patients were eliminated from the research according to the exclusion criteria as follows: patients with a second tumor before surgery (2 cases), patients who received neo-adjuvant chemotherapy (1 case), patients without R0 resection (1 case), lost follow-up (6 cases), the number of dissected lymph nodes was less than 15 (2 cases). At last, 200 recurrent PDAC patients were enrolled in the present study. Subsequently, the 200 patients were divided into a training cohort (132 cases) and a validation cohort (68 cases) by the 200 recurrent PDAC patients were divided into training and validation groups by LOOCV. For the training cohort, the median PPFS was 5.25 months. For the validation cohort, the median PPFS was 5.25 months. The clinicopathological characteristics of the two groups were established in Table 1. Based on the results of the chi-square test, T stage, TNM stage, tumor differentiation, chemotherapy after recurrence, and recurrence patterns showed a significant difference between the training and validation cohorts.

**Table 1:** Clinicopathological characteristics of PDAC patients in the training and validation cohort.

Characteristic	Training, *N* = 132	Validation, *N* = 68	*p*-value
Gender			0.7
Female	80 (61%)	43 (63%)	
Male	52 (39%)	25 (37%)	
Age			0.3
<60	42 (32%)	27 (40%)	
≥60	90 (68%)	41 (60%)	
Tumor site			>0.9
Head	103 (78%)	53 (78%)	
Body and Tail	29 (22%)	15 (22%)	
T stage			**<0.001**
T1	7 (5.3%)	14 (21%)	
T2	54 (41%)	14 (21%)	
T3	71 (54%)	40 (59%)	
N stage			0.064
N0	28 (21%)	19 (28%)	
N1	51 (39%)	33 (49%)	
N2	53 (40%)	16 (24%)	
TNM stage			**0.033**
I	15 (11%)	12 (18%)	
II	64 (48%)	41 (60%)	
III	53 (40%)	15 (22%)	
Tumor differentiation			**0.005**
Well-Moderate	34 (26%)	31 (46%)	
Poor	98 (74%)	37 (54%)	
Adjacent organ invasion			0.2
Absence	52 (39%)	21 (31%)	
Presence	80 (61%)	47 (69%)	
Microvascular invasion			0.4
Absence	67 (51%)	39 (57%)	
Presence	65 (49%)	29 (43%)	
Lymph vascular invasion			0.12
Absence	85 (64%)	36 (53%)	
Presence	47 (36%)	32 (47%)	
Perineural invasion			0.5
Absence	27 (20%)	17 (25%)	
Presence	105 (80%)	51 (75%)	
Chemotherapy after surgery			>0.9
Untreated	52 (39%)	25 (37%)	
Single-agent therapy	57 (43%)	31 (46%)	
Multi-agent therapy	23 (17%)	12 (18%)	
Chemotherapy after recurrence			**<0.001**
Untreated	47 (36%)	15 (22%)	
Single-agent therapy	29 (22%)	37 (54%)	
Multi-agent therapy	56 (42%)	16 (24%)	
Recurrence patterns			**0.006**
Local	13 (9.8%)	18 (26%)	
Lung only	29 (22%)	9 (13%)	
Liver only	35 (27%)	22 (32%)	
Local and distant	55 (42%)	19 (28%)	
Time to recurrence			0.9
Early recurrence	89 (67%)	45 (66%)	
Late recurrence	43 (33%)	23 (34%)	
CA199 (U/ml)			0.5
<35	31 (23%)	19 (28%)	
≥35	101 (77%)	49 (72%)	
CEA (ng/ml)			0.8
<5	61 (46%)	33 (49%)	
≥5	71 (54%)	35 (51%)	
NLR			0.078
<224.65	38 (29%)	28 (41%)	
≥224.65	94 (71%)	40 (59%)	
PLR			0.3
<2.28	88 (67%)	50 (74%)	
≥2.28	44 (33%)	18 (26%)	

The bold values represents *p*-values less than 0.05.

### Prognostic factors for PPFS

As shown in Table 2, 18 factors were enrolled into univariate Cox regression in the training cohort. As the results showed, 11 factors were described to be correlated with PPFS: tumor differentiation, T stage, N stage, time to recurrence, CA19-9, NLR, adjacent organ invasion, lymph vascular invasion, recurrence pattern, chemotherapy after surgery, chemotherapy after recurrence. Consequently, these factors were enrolled into the multivariate Cox regression, and the results were established in Table 3. The T stage, N stage, time to recurrence, Lymph vascular invasion, recurrence pattern, chemotherapy after surgery, chemotherapy after recurrence were considered as independent prognostic factors for PPFS in recurrent PDAC patients.

**Table 2:** The results of univariate cox regression in the training cohort.

Characteristics	HR	*p*	CI	Characteristics	HR	*p*	CI
Gender				Perineural invasion			
Male	Ref			Absence	Ref		
Female	0.86	0.412	0.59 – 1.24	Presence	1.18	0.473	0.75 – 1.83
Age				Microvascular invasion			
<60	Ref			Absence	Ref		
≥60	1.01	0.956	0.68 – 1.5	Presence	1.1	0.603	0.77 – 1.58
Tumor site				Adjacent organ invasion			
Head	Ref			Absence	Ref		
Body and Tail	1.28	0.264	0.83 – 1.97	Presence	1.47	**0.044**	1.01 – 2.14
Tumor differentiation				Lymph vascular invasion			
Well-Moderate	Ref			Absence	Ref		
Poor	1.68	**0.019**	1.09 – 2.59	Presence	1.54	**0.026**	1.05 – 2.25
T stage				Recurrence patterns			
T1	Ref			Local	Ref		
T2	2.85	**0.019**	1.19 – 6.81	Lung only	3.42	**0.011**	1.32 – 8.86
T3	3.47	**0.005**	1.47 – 8.17	Liver only	3.64	**0.007**	1.41 – 9.39
N stage				Local and distant	3.31	**0.011**	1.32 – 8.31
N0	Ref			Chemotherapy after surgery			
N1	1.51	0.108	0.91 – 2.51	Untreated	Ref		
N2	2.19	**0.002**	1.34 – 3.57	Single-agent therapy	0.65	**0.035**	0.44 – 0.97
Time to recurrence				Multi-agent therapy	0.45	**0.005**	0.26 – 0.78
Early recurrence	Ref			Chemotherapy after recurrence			
Late recurrence	0.61	**0.020**	0.41 – 0.92	Untreated	Ref		
CA199				Single-agent therapy	0.63	0.081	0.38 – 1.06
<35	Ref			Multi-agent therapy	0.54	**0.003**	0.36 – 0.82
≥35	1.64	**0.033**	1.04 – 2.59				
CEA							
<5	Ref						
≥5	1.27	0.214	0.87 – 1.85				
PLR							
<2.28	Ref						
≥2.28	0.91	0.629	0.61 – 1.35				
NLR							
<224.65	Ref						
≥224.65	1.57	**0.036**	1.03 – 2.38				

The bold values represents *p*-values less than 0.05.

**Table 3:** The results of multivariate cox regression in the training cohort.

Characteristics	HR	*p*	CI	Characteristics	HR	*p*	CI
Tumor differentiation				Adjacent organ invasion			
Well-Moderate	Ref			Absence	Ref		
Poor	1.09	0.753	0.64 – 1.87	Presence	0.99	0.9613	0.63 – 1.56
T stage				Lymph vascular invasion			
T1	Ref			Absence	Ref		
T2	3.23	**0.017**	1.24 – 8.42	Presence	4.21	**0.001**	2.42 – 7.33
T3	3.86	**0.010**	1.39 – 10.71	Recurrence patterns			
N stage				Local	Ref		
N0	Ref			Lung only	4.77	**0.004**	1.65 – 13.82
N1	2.43	**0.007**	1.28 – 4.62	Liver only	5.02	**0.002**	1.79 – 14.06
N2	2.59	**0.002**	1.43 – 4.7	Local and distant	5.11	**0.002**	1.84 – 14.18
Time to recurrence				Chemotherapy after surgery			
Early recurrence	Ref			Untreated	Ref		
Late recurrence	0.54	**0.035**	0.3 – 0.96	Single-agent therapy	0.64	0.058	0.4 – 1.02
CA199				Multi-agent therapy	0.36	**0.003**	0.18 – 0.7
<35	Ref			Chemotherapy after recurrence			
≥35	1.18	0.559	0.68 – 2.07	Untreated	Ref		
NLR				Single-agent therapy	0.57	0.058	0.32 – 1.02
<224.65	Ref			Multi-agent therapy	0.48	**0.002**	0.3 – 0.77
≥224.65	0.67	0.169	0.38 – 1.18				

The bold values represents *p*-values less than 0.05.

### Survival analysis for independent prognostic factors

**Figure 2:**
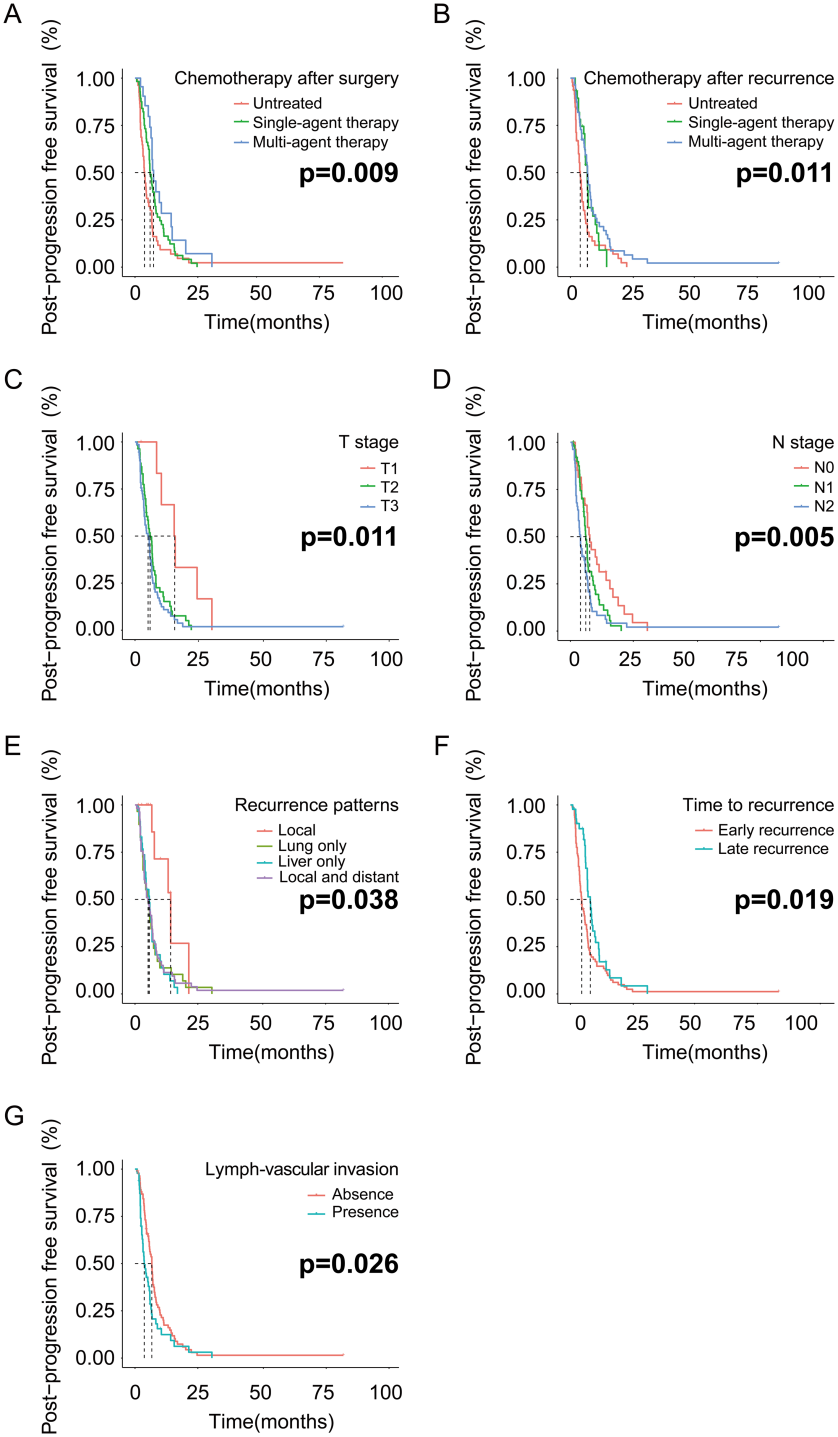
The PPFS analysis based on the independent risk factors in the training cohort. **(A)** PPFS analysis based on chemotherapy after surgery; **(B)** PPFS analysis based on chemotherapy after recurrence; **(C)** PPFS analysis based on T stage; **(D)** PPFS analysis based on N stage. **(E)** PPFS analysis based on recurrence patterns; **(F)** PPFS analysis based on time to recurrence; **(G)** PPFS analysis based on lymph vascular invasion.

The patients who received multi-agent therapy showed better PPFS than patients treated with single-agent therapy (*p* = 0.009) (Figure 2A). Meanwhile, the patients treated with multi-agent or single-agent chemotherapy regiments after tumor relapse were estimated to have a similar prognosis (*p* = 0.011) (Figure 2B). The PDAC patients in the T1 stage had a finer prognosis than patients in the T2 or T3 stage (*p* = 0.011) (Figure 2C). Worse outcomes are observed in patients with higher N stages (*p* = 0.005) (Figure 2D). The patients with local recurrence had the best prognosis among the four relapse patterns mentioned in this study (*p* = 0.038) (Figure 2E). PDAC patients with late recurrence had a relatively better prognosis than early recurrent patients (*p* = 0.019) (Figure 2F). Finally, positive lymph vascular invasion in PDAC patients predicted worse outcomes when compared with negative lymph vascular invasion (*p* = 0.026) (Figure 2G).

### Construction and validation of a nomogram for PPFS prediction

**Figure 3:**
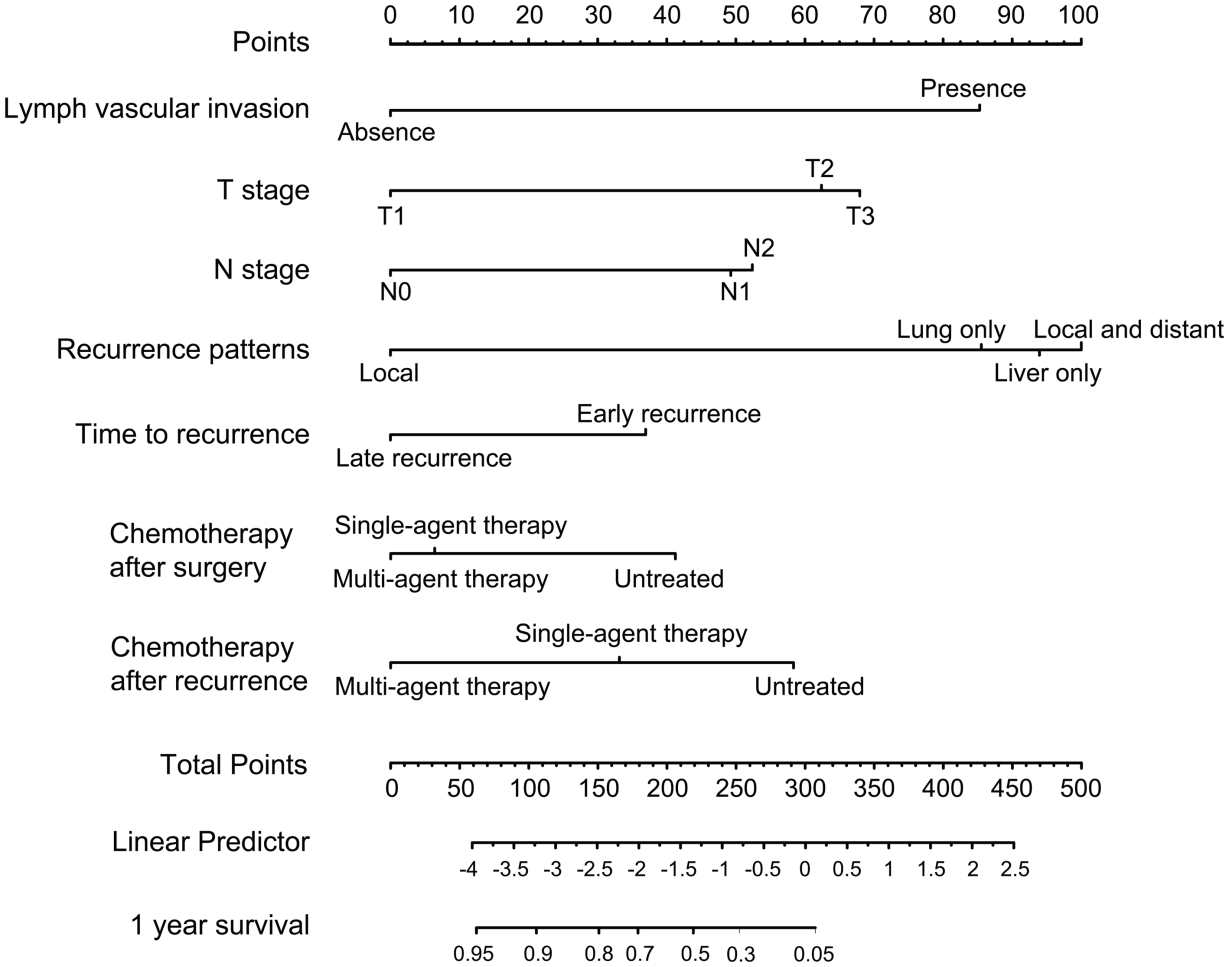
Nomogram for predicting the 1-year PPFS rates of PDAC patients after radical resection in the training cohort.

**Figure 4:**
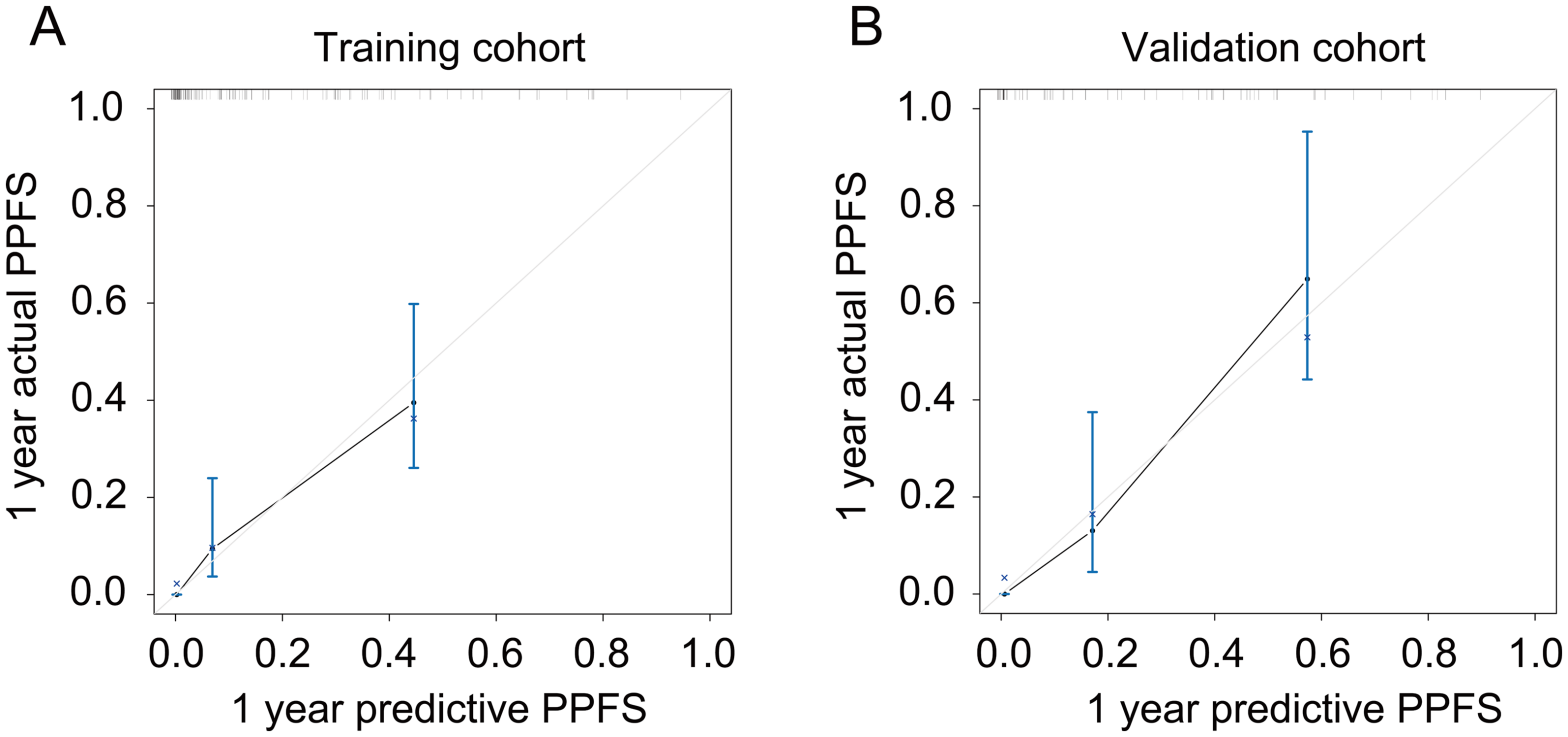
The calibration plot for predicting PPFS rates in recurrent PDAC. **(A)** The calibration plot for predicting the 1-year PPFS rates in the training cohort; **(B)** The calibration plot for predicting the 1-year PPFS rates in the validation cohort.

**Figure 5:**
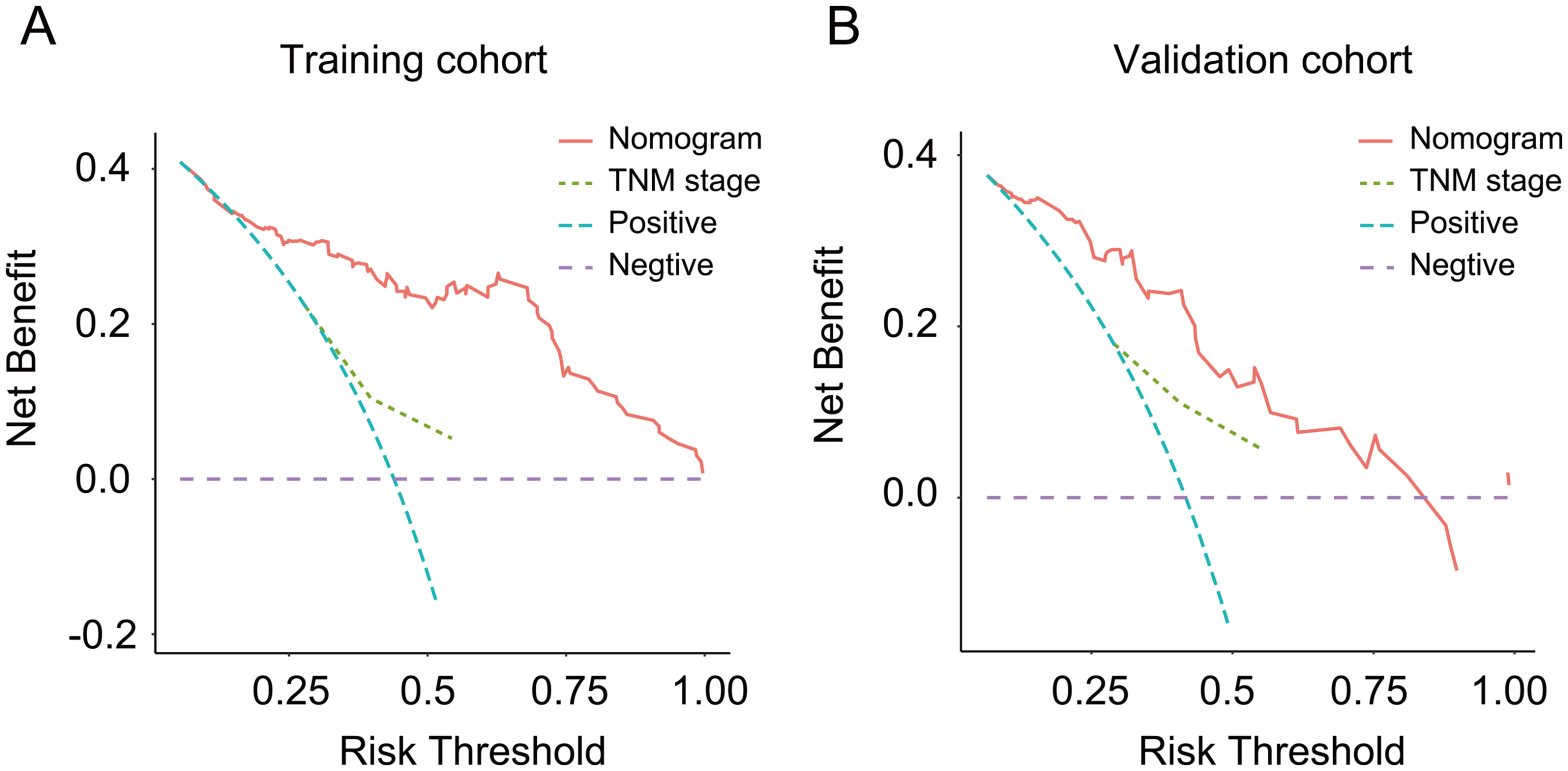
The decision curve analysis for the nomogram and TNM stage. **(A)** Decision curve analysis for the nomogram and TNM stage in the training cohort; **(B)** decision curve analysis for the nomogram and TNM stage in the validation cohort.

As shown in Figure 3, a specific nomogram for PPFS prediction in patients with PDAC was built on independent prognostic factors. The recurrence patterns are the factor that displayed the most prominent effect in this model, followed by the lymph vascular invasion, T-stage, N-stage, chemotherapy after recurrence, chemotherapy after surgery, and time to recurrence. Furthermore, to detect the accuracy of the nomogram model, the calibration plots were produced, demonstrating a high conformity between the actual and predictive PPFS in both training and validation groups (Figure 4). Meanwhile, to assess the discriminatory ability between the nomogram and TNM stage system, the C-index was calculated based on the training cohort and validation cohort; as shown in Table 4, the C-index of the nomogram model was significantly higher than that in the TNM stage in both training and validation cohort. Finally, the decision curve analysis was fabricated to compare the clinical benefits of the nomogram and the TNM stage system (Figure 5). It means that the nomogram built in the present study could be more beneficial for clinical prediction than the TNM stage system.

**Table 4:** Comparison of the C-index between nomogram and TNM stage.

Cohort	Model	C-index
Validation cohort	Nomogram	0.739
	TNM stage	0.726
Training cohort	Nomogram	0.609
	TNM stage	0.596

## Discussion

PDAC was a lethal disease in which micro-metastatic lesions occurred in the early stage of carcinogenesis (22). Therefore, nearly 85% of the PDAC patients who received radical surgery would suffer from tumor relapse in the early postoperative period (6, 7). Tumor recurrence was portrayed as a crucial time point for PDAC patients since 80% of them may die from tumor recurrence or metastasis during the next five years (23, 24). Moreover, the advancement of recurrent lesions was also demonstrated as another primary concern, from which the alternative chemotherapy regimen should be considered for restricting the growth of recurrent lesions (13, 25). Therefore, exploring the factors independently influencing the PPFS and precisely predicting the PPFS through the nomogram model was essential for developing alternative chemotherapy regimens and improving patient outcomes. However, the correlation between the clinicopathological factors and PPFS has not been explored clearly. The present study determined that lymph vascular invasion, N stage, T stage, recurrence patterns, time to recurrence, chemotherapy after surgery, and chemotherapy after recurrence were independent prognostic factors for PPFS. Furthermore, we also constructed a nomogram based on those independent prognostic factors with favorable predictive capacity.

Since the high invasive capacity of PDAC, micro-metastatic lesions and residual tumor foci commonly co-existed in the same patient (22, 26, 27), adjuvant chemotherapy was imminent after radical resection (28–31). The preceding research declared that chemotherapy inhibited tumor progression and metastasis (32–35). However, the tumor-inhibiting effect of chemotherapy on the relapse lesions had been rarely discussed before. The present study found that PDAC patients received multi-agent chemotherapy after surgery showed better PPFS than patients received single-agent chemotherapy. Meanwhile, we also found that multi-agent chemotherapy and single-agent chemotherapy had similar efficacy in limiting the progression of relapse foci. Drug-tolerant tumor cells was fundamental for drug-resistant clones and contribute to relapse and disease progression (36). It means that the resistance to chemotherapy was significantly strengthened in the recurrent lesion compared with the primary tumor. Meanwhile, the progression of recurrent lesions was too fast to be restricted by the chemotherapeutic agent (37, 38). Therefore, the superiority of the multi-agent regimen was masked when compared with the single-agent schema in recurrent PDAC patients (39–43). Based on the results of the present study, we argue that multi-agent chemotherapy brings more survival benefits to PDAC patients after radical surgery compared with a single-agent scheme. However, single-agent chemotherapy regimens should also be recommended for recurrent PDAC patients with poor chemotherapy tolerance to reduce the toxic side effects of chemotherapeutic agents (44, 45).

In previous research, lymph node metastasis was explained as an essential predictor for tumor progression (46–49). Meanwhile, the consensus in the Japanese Pancreatic Society also cited that confirmation of N9 and N16 lymph node metastasis was intimately linked with tumor relapse and distant metastasis (14). Similarly, the higher N stage and positive lymph vascular invasion portended early advancement of recurrent lesions in the present research. Dissemination into the lymphatic system was the major routes for PDAC metastasis (50). Meanwhile, lymph node metastasis was an initial step in PDAC metastasis. It was also crucial for determination of clinical staging, prognosis and survival in PDAC patients (51, 52). Therefore, recurrent PDAC patients with lymph vascular invasion or lymph node metastasis may benefit from chemotherapy for inhibiting the relapse lesion progression.

The T stage represented the tumor diameters measured by the pathologists, indicating the tumor burden. Moreover, it has also been estimated as the reference standard for chemotherapeutic efficacy (13, 53, 54). The probability that a cancer contains drug-resistant clones depended on the size of the tumor (55). In this study, we found that the higher T stage was one of the independent prognostic factors for PPFS, portending a shorter PPFS. The residual disease of PDAC patients after surgery my cause tumor recurrence or relapse lesions progression. Hence, the radiotherapy or nano knife against the resection margin may reduce the local recurrence rate in PDAC patients with large tumor volumes.

Different recurrence patterns of PDAC patients are usually linked with diverse post-progression survival (7, 21, 56). In the earlier study, the local and distant recurrence pattern heralded the poorest post-progression survival among the four abovementioned recurrence patterns (7). The present study also estimated that the local and distant recurrence patterns predicted poorer PPFS compared to the local recurrence patterns. The late tumor stage may be one of the most important causes leading to this result (7, 57). Therefore, PDAC patients with local and distant recurrence are warranted to received chemotherapy when compared with local recurrent cases.

Last but not least, we investigated the relationship between the time to recurrence and PPFS. The results declared that PDAC patients with late relapse had exclusively longer PPFS compared with the early relapse patients. In the previous research, early recurrence reflected the malignant phenotype of PDAC (8, 14, 58). Most of the early relapse patients were combine with late tumor stage, poorer tumor differentiation, and stronger drug-resistant effect, causing a more rapid progression of recurrent lesions (7, 59, 60). Therefore, chemotherapy-only may be not enough to restrain the tumor progression in early recurrent PDAC patients. Combination therapies may be more suitable for early recurrent cases. In detail, secondary surgery could be performed in PDAC patients with isolated local recurrent or oligometastatic lesions. Besides this, radiofrequency ablation or radiation therapy combined with chemotherapy could be another option for early recurrent patients with multiple metastases.

The development of recurrent lesions was a crucial time point for replacing chemotherapy schemes according to the NCCN guidelines (13). Furthermore, individualized intervention for PDAC patients should be managed based on the risk of relapse lesions advancement. Therefore, precise prediction for PPFS is essential for clinical decision-making. After statistical analysis, our research selected several independent prognostic factors from high-dimensional radiological and pathological variables. Furthermore, we also constructed a nomogram for PPFS prediction based on those independent prognostic factors. The results of contrast analysis (the calibration curve, DCA, and C-index) between the training and validation cohort demonstrated this nomogram system’s strong predictive and discriminative power. Thus, clinicians can precisely assess the risk of progression in relapse lesions for PDAC patients by using the nomogram system fabricated in this study.

There are still some limitations to the present study. First, this study adopted a single-center retrospective design. The regional difference of clinical practices might affect the extrapolation of the results in our current analysis. A multi-center control study was needed to better estimate the predictive capacity of the nomogram constructed in the present study. Meanwhile, a major potential bias in this analysis is selection bias. For example, there was a lower readmission probability in the PDAC patients with history of stroke or coronary artery disease because of the higher surgical risk when compared with PDAC patients with better physical condition. It may limit the predictive power of the nomogram in the PDAC patients with poor physical status.

Hence, to minimize such bias, we inflated the sample size by including the resected PDAC patients from 2008 to 2019, and we also set strict inclusion criteria and exclusion criteria for screening. On the other hand, some variables, such as serum total bilirubin levels, C-reactive protein, and albumin, had not been included in this research. It was beneficial to explore the impact of nutritional status and inflammatory response on the progression of relapse lesions in PDAC patients. Meanwhile, some of the characteristics were only vaguely described during the information collection. For example, the specific chemotherapy regimens and the lymph node metastasis group had not been displayed in detail. The involvement of the characteristics mentioned above could further refine the predictive effect of the nomogram. For instance, more precise results would be established in future exploration when a larger sample size was available. Finally, some modified described methods for lymph node metastasis, such as lymph node ratio and log odds of positive lymph nodes, have already been proposed in some research (61). However, we still referred to the description of lymph node metastasis from the AJCC guidelines in the present study. More persuasive results would be available if future research could apply the modified methods to lymph node metastasis discrimination.

## Conclusion

Multi-agent chemotherapy is recommended for PDAC patients after surgery. The single-agent chemotherapy also deserves consideration after tumor recurrence. The nomogram model provided a new way for PPFS prediction in recurrent PDAC patients. Moreover, the predictive value and accuracy of the present nomogram will be improved if validated in a larger and multi-center cohort.

## Data Availability

The datasets presented in this study can be found in online repositories. The names of the repository/repositories and accession number(s) can be found in the article/Supplementary material.
